# Cellular internalization of alpha-synuclein aggregates by cell surface heparan sulfate depends on aggregate conformation and cell type

**DOI:** 10.1038/s41598-017-08720-5

**Published:** 2017-08-21

**Authors:** Elisabet Ihse, Hodaka Yamakado, Xander M. van Wijk, Roger Lawrence, Jeffrey D. Esko, Eliezer Masliah

**Affiliations:** 1 Department of Neurosciences, University of California, San Diego, La Jolla, CA 92093 USA; 2 Department of Cellular and Molecular Medicine, Glycobiology Research and Training Center, University of California, San Diego, La Jolla, CA 92093 USA; 3 Department of Laboratory Medicine, University of California, San Francisco, USA

## Abstract

Amyloid aggregates found in the brain of patients with neurodegenerative diseases, including Alzheimer’s and Parkinson’s disease, are thought to spread to increasingly larger areas of the brain through a prion-like seeding mechanism. Not much is known about which cell surface receptors may be involved in the cell-to-cell transfer, but proteoglycans are of interest due to their well-known propensity to interact with amyloid aggregates. In this study, we investigated the involvement of plasma membrane-bound heparan and chondroitin sulfate proteoglycans in cellular uptake of aggregates consisting of α-synuclein, a protein forming amyloid aggregates in Parkinson’s disease. We show, using a pH-sensitive probe, that internalization of α-synuclein amyloid fibrils in neuroblastoma cells is dependent on heparan sulfate, whereas internalization of smaller non-amyloid oligomers is not. We also show that α-synuclein fibril uptake in an oligodendrocyte-like cell line is equally dependent on heparan sulfate, while astrocyte- and microglia-like cell lines have other means to internalize the fibrils. In addition, we analyzed the interaction between the α-synuclein amyloid fibrils and heparan sulfate and show that overall sulfation of the heparan sulfate chains is more important than sulfation at particular sites along the chains.

## Introduction

Protein aggregation is a hallmark of many neurodegenerative diseases, including Alzheimer’s disease and Parkinson’s disease^1^. A large body of evidence demonstrates that protein aggregation is not an epiphenomenon, but rather drives disease development^2^. The specific proteins that aggregate and form deposits vary between different neurodegenerative diseases, but the aggregates often share a similar beta-sheet rich fold, forming long unbranched structures called amyloid fibrils^3, 4^. A well-known propensity of amyloid fibrils is to act as auto-catalysts, triggering further incorporation of monomeric protein into the fibrils, a process known as “seeding”^5, 6^. Evidence also indicates that spreading of protein aggregation to increasingly larger areas of the brain and the ensuing pathological changes are caused by a seeding mechanism^7–15^.

In Parkinson’s disease (PD) and dementia with Lewy Bodies (DLB), the hallmark deposits (Lewy bodies and Lewy neurites) are predominantly found inside neurons. The fibril forming protein in these deposits is α-synuclein, a 14 kDa presynaptic protein^2^. Alpha-synuclein aggregates are also seen in oligodendrocytes in multiple system atrophy (MSA)^16^. Lewy bodies have been found in grafted neurons in Parkinson’s disease patients treated with embryonic cell transplants^7^. In addition, animal studies have shown that brain inoculation with α-synuclein aggregates, or over-expression of α-synuclein in restricted brain areas, lead to propagation of α-synuclein aggregation to anatomically interconnected areas of the brain^9, 10, 14, 15, 17^. Cell culture studies have shown that cells internalize α-synuclein aggregates, and that once inside, the aggregates can trigger further aggregation of intracellular α-synuclein^8, 9, 11^. However, the molecular actors and pathways involved in both secretion and internalization remain obscure.

Proteoglycans are glycoproteins that contain one or more sulfated glycosaminoglycan (GAG) chains^18^. Cell surface proteoglycans are found on virtually all animal cells. They bind a number of protein ligands, and are indispensable during embryonic development and organ physiology^18, 19^. GAGs, in particular heparan sulfate, interact with amyloid proteins^20–30^. The interaction likely occurs by way of negatively charged groups in the GAG chains with positively charged amino acids in the amyloid protein^19, 31^. Heparan sulfate has been found in all extracellular amyloid deposits investigated, regardless of the nature of the amyloid protein^20, 21^. In addition, heparan sulfate induces fibril formation of many amyloidosis-related proteins *in vitro* ^22–26^, and also has a stabilizing effect on the formed aggregates^32^. More recently, cell surface heparan sulfate proteoglycans have been described to facilitate cellular internalization of amyloid proteins, which has attracted attention due to the emerging interest in disease propagation through cell-to-cell transfer of amyloid aggregates^27–30^.

Here, we examined the involvement of GAGs in cellular internalization of α-synuclein aggregates. We show that in neuronal cells, internalization of α-synuclein in the form of amyloid fibrils depends on heparan sulfate, whereas α-synuclein in the form of soluble non-amyloid oligomers does not. We show that uptake of α-synuclein amyloid fibrils in oligodendrocytic cells also depends on heparan sulfate, while it is less important for astrocytic and microglial uptake. Lastly, we investigated how the extent and pattern of heparan sulfate sulfation affects cellular uptake of α-synuclein fibrils.

## Results

### Formation and characterization of two different α-synuclein aggregate species

**Figure 1:**
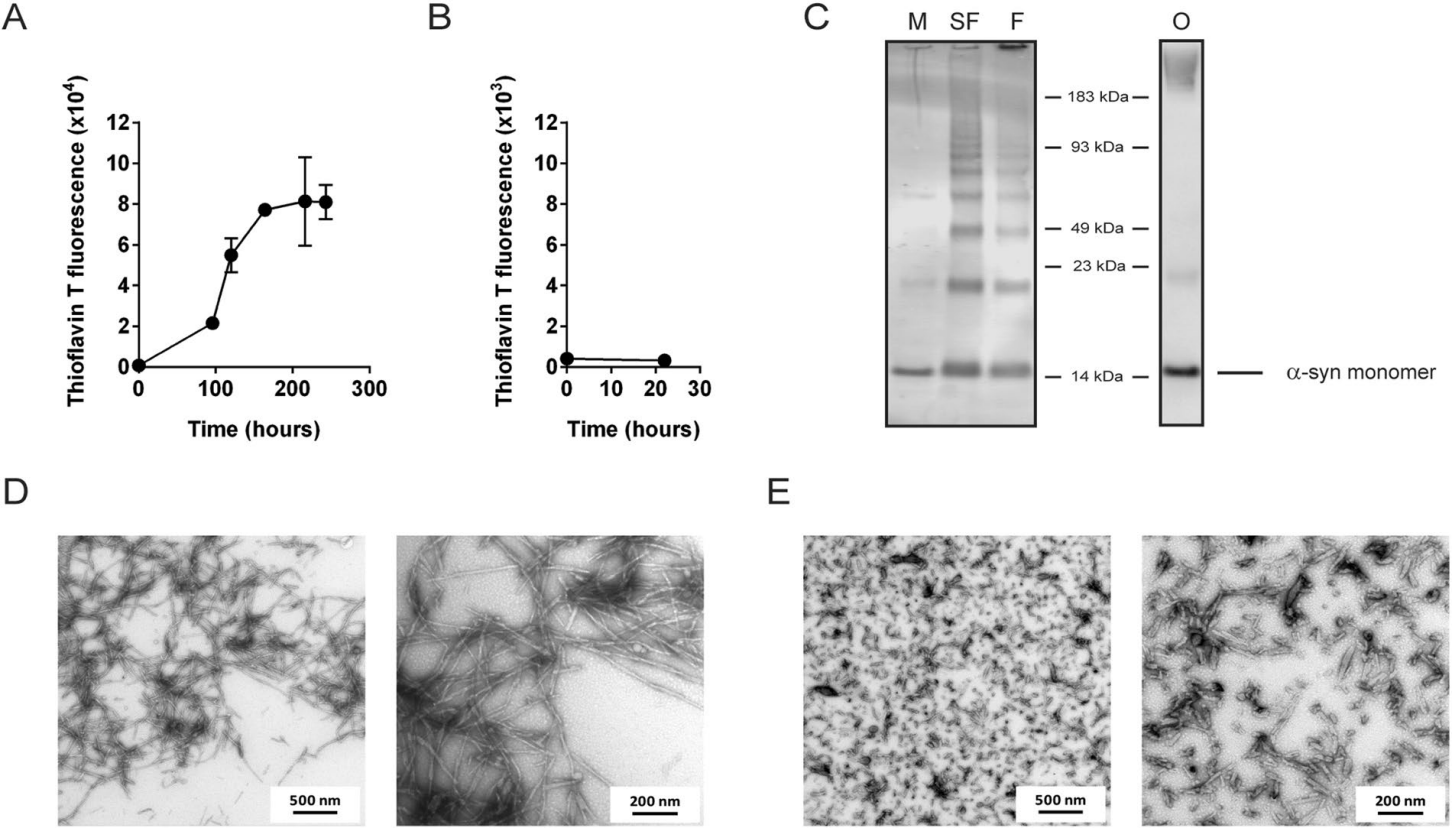
Characterization of α-synuclein aggregates. (**A**) Amyloid fibril formation was followed by a Thioflavin T fluorescence assay. Fibril formation was stopped after 10 days, when the Thioflavin T fluorescence had reached its plateau. (**B**) The oligomeric preparation showed no increase in Thioflavin T fluorescence. (**C**) Western blot analysis of α-synuclein preparations using an anti-synuclein antibody (FL-140, Santa Cruz). M = Monomers, SF = Sonicated fibrils, F = Fibrils, without sonication, O = Oligomers. (**D**,**E**) Electron microscopy on the fibril preparation showed typical amyloid fibrils (**D**) that broke into shorter fibrils after sonication (**E**).

To examine if the particular conformation of α-synuclein aggregates would affect uptake into cells by way of heparan sulfate proteoglycans, we generated two α-synuclein aggregate species - soluble non-amyloid oligomers and fibrils with a typical amyloid fold. Monomeric recombinant human α-synuclein was used to produce the aggregates and the conformation was verified by Thioflavin T fluorescence, western blot and electron microscopy (Fig. 1). The amyloid fibrils were sonicated before they were added to the cell culture. Sonication breaks the fibrils into shorter structures and separates the fibrils from each other, thereby ensuring that the preparation does not contain large networks of fibrils whose size exceed what the cells are able to internalize. In addition, sonicated α-synuclein fibrils have been shown to be able to act as seeds for further α-synuclein aggregation^11, 14, 17, 33, 34^. Such species may therefore be of particular interest when studying cellular internalization of protein aggregates.

### Binding of α-synuclein fibrils, but not soluble non-amyloid oligomers, depends on heparan sulfate in neuroblastoma cells

**Figure 2:**
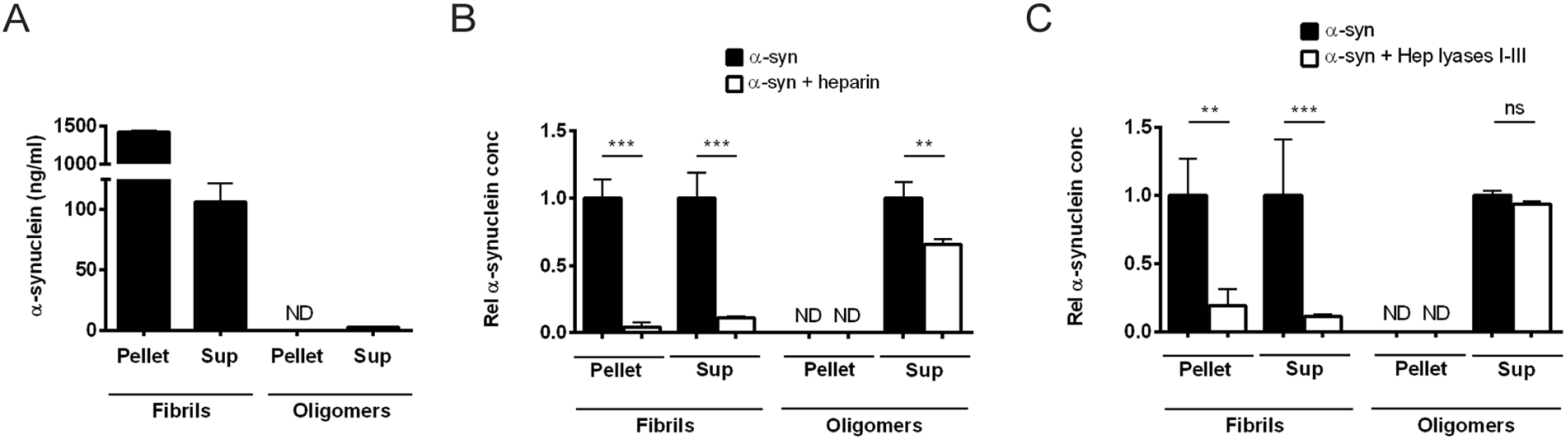
Cellular binding/uptake of α-synuclein amyloid fibrils, but not soluble non-amyloid oligomers, depends on heparan sulfate in B103 cells. B103 neuroblastoma cells were harvested 4 hr after addition of α-synuclein to the cell media, and cell lysates were centrifuged and separated into pellet and supernatant fractions. Each fraction was analyzed for α-synuclein content by a sandwich ELISA. The vast majority of fibrillar α-synuclein was, as expected, found in the cell lysate pellet while the non-amyloid oligomeric α-synuclein was only found in the cell lysate supernatant. (**A**) Cell binding/uptake of α-synuclein amyloid fibrils and soluble non-amyloid oligomers. (**B**) Binding/uptake of α-synuclein of cells treated with heparin (20 µg/ml) prior to addition of α-synuclein. (**C**) Binding/uptake of α-synuclein of cells pre-treated with heparin lyases (mixture of I, II and III, 5 mU/ml) prior to addition of α-synuclein. ND = not detected. Statistical significance was analyzed by one-way ANOVA with Sidak’s multiple comparisons test.

To examine the binding and uptake of the different α-synuclein preparations, fibrils and oligomers were added to rat neuroblastoma B103 cells. After four hours, the cells were solubilized and centrifuged, and α-synuclein in the pellet and supernatant fractions was measured by sandwich ELISA. High binding/uptake was seen for the fibrillar preparation. As expected, most of the α-synuclein in this preparation was recovered in the pellet, although some was found in the supernatant, possibly due to smaller aggregate species generated by sonication of the fibrils (Fig. 2A). In comparison, very little soluble non-amyloid oligomers were bound or taken up. These oligomeric species were only present in the supernatant, while no α-synuclein could be detected in the pellet (Fig. 2A). Addition of heparin (a highly sulfated variant of heparan sulfate) almost eliminated all cellular binding/internalization of α-synuclein amyloid fibrils, by competing with cell surface heparan sulfate. The same treatment had little effect on the soluble non-amyloid oligomers (Fig. 2B). Pre-treating the cells with a mixture of the heparan sulfate degrading enzymes heparin lyases I, II and III also decreased the binding/uptake of fibrillar α-synuclein to a great extent, but had very little effect on oligomers (Fig. 2C).

These results suggested that the association of α-synuclein fibrils to B103 cells depend on cell surface heparan sulfate. Interestingly, the heparan sulfate involvement was limited to aggregates with an amyloid fibrillar structure, while the soluble non-amyloid oligomers did not seem to depend on the presence of these cell surface molecules.

### Alpha-synuclein amyloid fibrils colocalize with heparan sulfate in neuroblastoma cells

**Figure 3:**
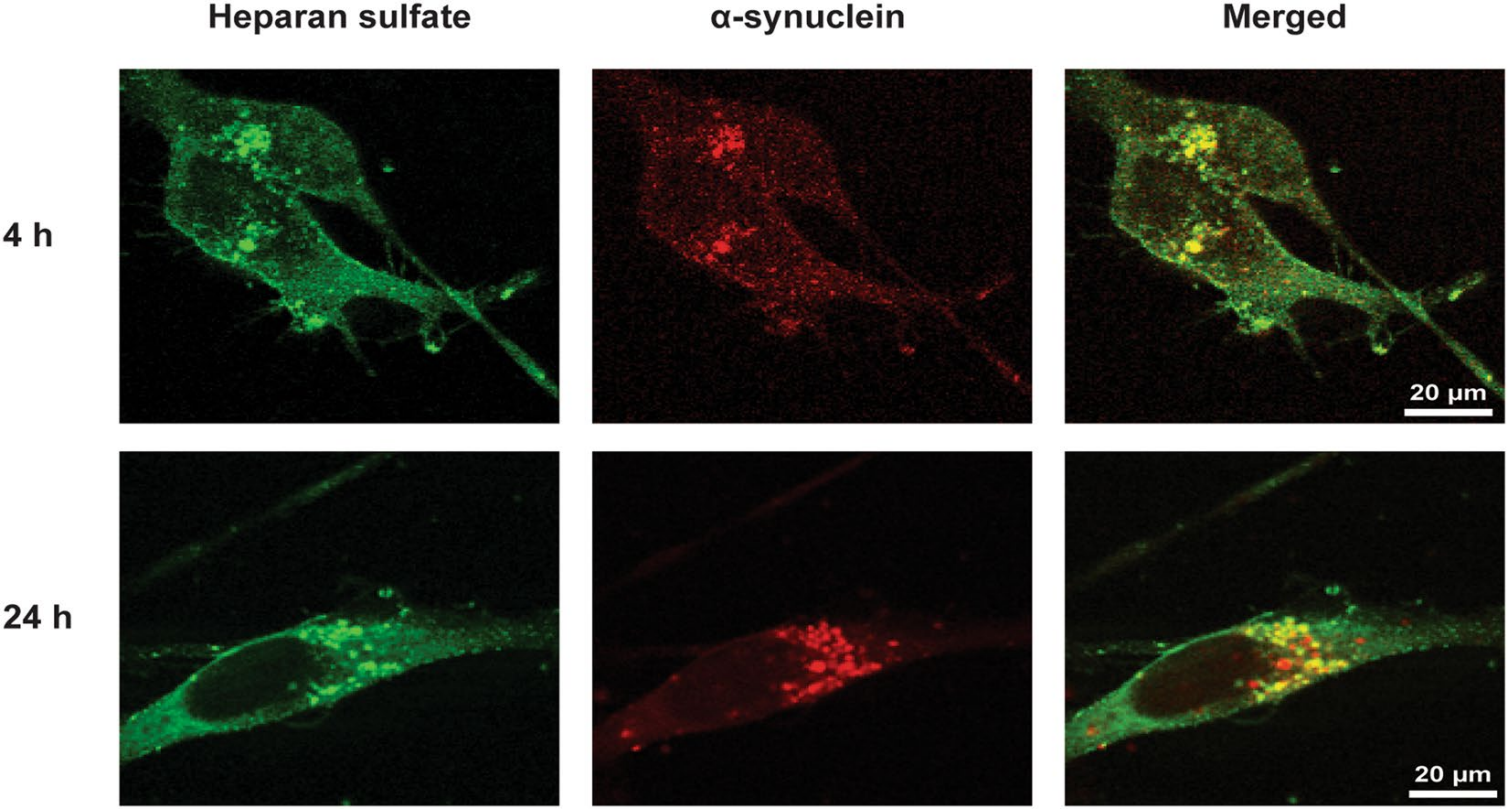
Colocalization of α-synuclein amyloid fibrils and heparan sulfate in B103 cells. Alpha-synuclein fibrils were labeled with AlexaFluor594 and added to the cell media. Cells were fixed at various time-points after α-synuclein addition, and thereafter stained with the anti-heparan sulfate antibody 10E4 and visualized with confocal microscopy.

Based on these findings, we predicted that α-synuclein amyloid fibrils should colocalize with heparan sulfate on the cell surface or within the endosomal/lysosomal pathway. B103 cells treated with AlexaFluor594 labeled α-synuclein fibrils and stained with a monoclonal antibody specific for heparan sulfate (10E4) demonstrated colocalization in numerous puncta after 4 hours (Fig. 3, upper panels). After 24 hours, the aggregates coalesced and increased in size (Fig. 3, lower panels).

### Cellular internalization of α-synuclein amyloid fibrils depends on heparan sulfate in neuroblastoma cells

**Figure 4:**
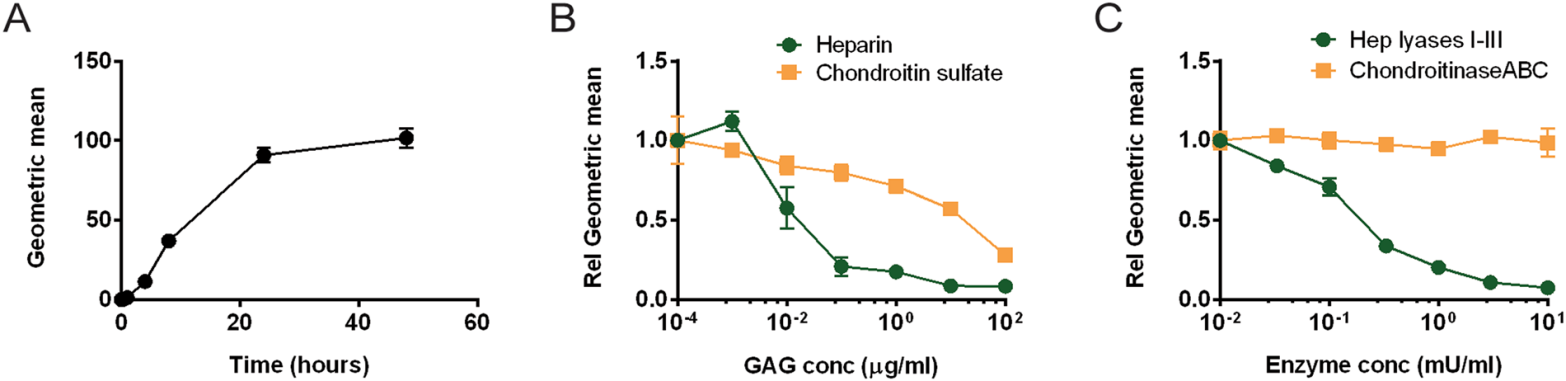
Internalization of α-synuclein amyloid fibrils in B103 cells depends on heparan sulfate. Alpha-synuclein fibrils labeled with pHrodo were added to B103 cells and internalization was measured by flow cytometry. (**A**) Time course of internalization of pHrodo-α-synuclein fibrils in B103 cells. (**B**,**C**) Cellular internalization of pHrodo-α-synuclein fibrils was determined after 8 hr of incubation **(B)** Dose-response curves when heparin or chondroitin sulfate was added to the cell media before addition of pHrodo-α-synuclein fibrils. (**C**) Dose-response curves when heparin lyases or chondroitinase ABC were added to the cell media before addition of pHrodo-α-synuclein fibrils.

The ELISA assay described above does not discriminate between α-synuclein bound to proteoglycans on the cell surface and material that has been internalized. To reduce the possibility of including cell-surface bound α-synuclein in the measurements, cells were treated with trypsin before harvesting. However, such treatment does not guarantee elimination of cell-surface bound amyloid fibrils, as these aggregates are known to be resistant to protease cleavage^35, 36^. To better assess cellular uptake, α-synuclein fibrils were labeled with the pH-sensitive dye pHrodo, which will only fluoresce with a strong signal at the acidic pH encountered in the endosomal-lysosomal pathway. B103 cells took up pHrodo-tagged α-synuclein in a time dependent way, saturating by 20 hours (Fig. 4A). The addition of heparin to the cell media decreased the uptake of pHrodo-α-synuclein fibrils in a dose-response manner with an IC_50_ of ~10 ng/ml (Fig. 4B). In contrast, inclusion of chondroitin sulfate had little effect at this concentration and had an approximate IC_50_ of ~10 µg/ml (Fig. 4B). Pre-treating cells with a mixture of heparin lyases I, II and III produced similar results as addition of heparin (Fig. 4C), whereas pre-treatment with chondroitinase ABC did not affect the uptake of α-synuclein amyloid fibrils (Fig. 4C). These observations demonstrate that cell surface heparan sulfate proteoglycans play a dominant role in uptake of α-synuclein fibrils.

### Cellular internalization of α-synuclein amyloid fibrils by heparan sulfate in glial cells

**Figure 5:**
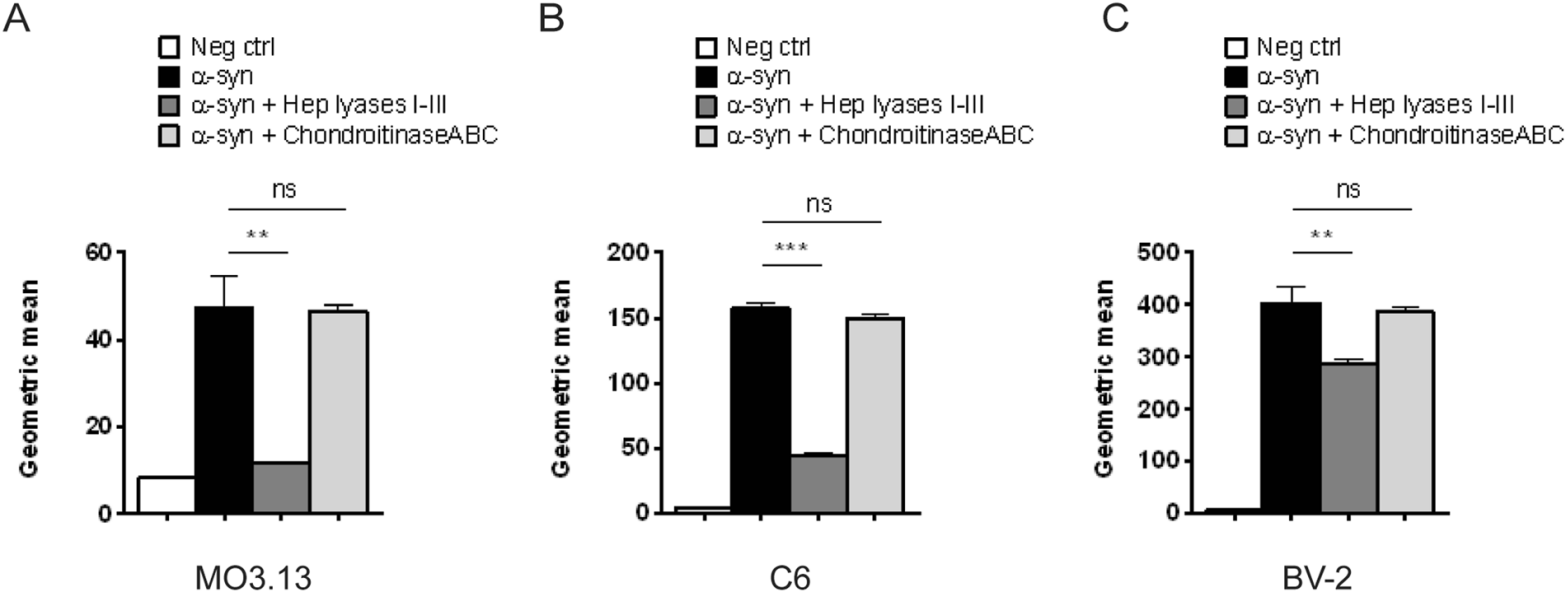
The involvement of heparan sulfate in internalization of α-synuclein fibrils in different types of glial cell lines. The cell lines were treated with a mix of heparin lyases I, II and III or chondroitinase ABC (5 mU/ml) before pHrodo-α-synuclein fibrils were added to the cell media. Internalization was measured by flow cytometry after incubation with pHrodo-α-synuclein fibrils for 8 hr. (**A**) Internalization of pHrodo-α-synuclein fibrils in the oligodendrocytic human MO3.13 cell line. (**B**) Internalization of pHrodo-α-synuclein fibrils in the astrocyte-like rat glioma C6 cell line. (**C**) Internalization of pHrodo-α-synuclein fibrils in the mouse microglial BV2 cell line. Statistical significance was analyzed by Student’s t-test.

To examine the dependence of α-synuclein uptake on heparan sulfate in other cell types found in the brain, we investigated several glial cell lines; the human oligodendrocytic cell line MO3.13, the astrocytic-like rat glioma C6 cell line and the murine microglial cell line BV-2. All three cell lines internalized pHrodo-tagged α-synuclein fibrils, but the degree of dependence on heparan sulfate differed, based on sensitivity of uptake to heparin lyase treatment (Fig. 5, dark grey bars). Uptake in MO3.13 cells was dramatically reduced by removal of heparan sulfate (Fig. 5A), similar to what was seen for the neuroblastoma B103 cells. In contrast, reduction in fibril uptake was less drastic in C6 cells (Fig. 5B), and uptake by BV-2 cells was only marginally affected (Fig. 5C). This suggests that the latter two cell types use other, heparan sulfate-independent, mechanisms to internalize the amyloid fibrils. Uptake was not affected by removal of chondroitin/dermatan sulfate via chondroitinase ABC treatment in any of the cell lines (Fig. 5, light gray bars).

### Characterization of heparan sulfate sulfation patterns important for cellular uptake of α-synuclein fibrils

The binding of protein ligands to heparan sulfate generally depends on electrostatic interactions between the negatively charged sulfate groups in the heparan sulfate chains with positively charged amino acids in the combining site in the heparan sulfate-binding protein^19, 31^. In some cases, binding depends on the overall degree of sulfation, including length of and distance between sulfate-rich domains. In other cases, binding depends on specific arrangements of sulfated disaccharide units (*N*-sulfated, 6-*O*-sulfated, and 3-*O*-sulfated glucosamine residues and 2-*O*-sulfated uronic acids). The characteristics of heparan sulfate required for its interaction with α-synuclein fibrils have not been determined.

**Figure 6:**
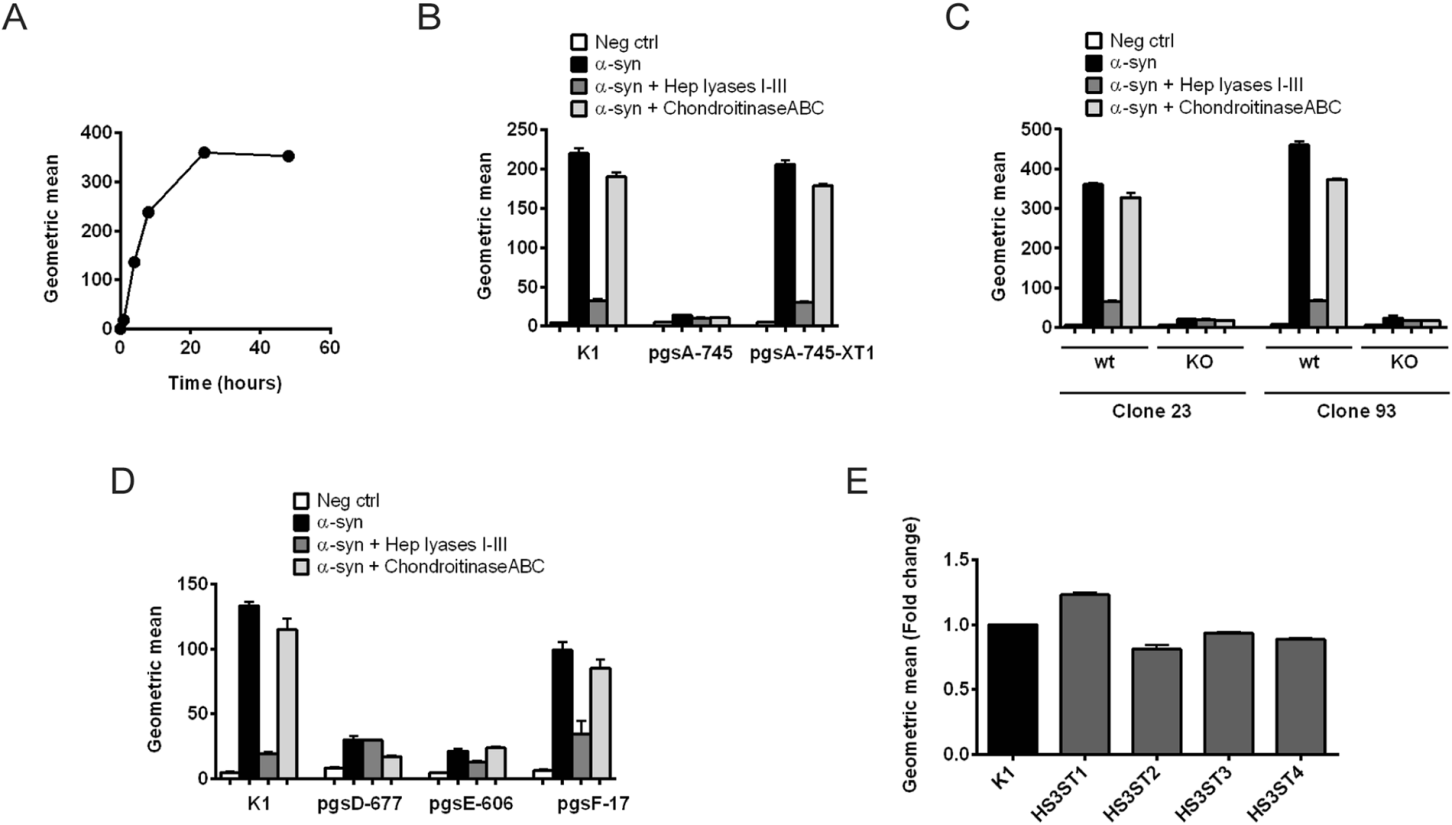
Internalization of α-synuclein fibrils by CHO cells deficient in different enzymes involved in GAG synthesis. pHrodo-α-synuclein fibrils were added to the cell media and their cellular internalization was analyzed with flow cytometry. Each cell line was treated with a mix of heparin lyases I, II and III or chondroitinase ABC (5 mU/ml) as a means to check the accuracy of the results obtained from the different lines. (**A**) Time course of pHrodo-α-synuclein fibrils internalization in CHO K1 (wt) cells. (**B**–**E**) Internalization of pHrodo-α-synuclein fibrils after incubation for 8 hr in CHO cells with different mutations. (**B**) Internalization of pHrodo-α-synuclein fibrils in CHO cells deficient in all GAGs (pgsA-745 strain), caused by insufficient xylosyltransferase activity, and in CHO pgsA-745 cells stably transfected with xylosyltransferase 1 (pgsA-745-XT1). (**C**) Internalization of pHrodo-α-synuclein fibrils in two different clones (clone 23 and 93) selected from CHO K1 cells, in comparison to the same clones where *XylT2* has been knocked out. (**D**) Internalization of pHrodo-α-synuclein fibrils in CHO cells deficient in enzymes involved in heparan sulfate synthesis. The pgsD-677 strain lacks HS, due to deficiency in Ext1, which is required for polymerization of the heparan sulfate chain. pgsE-606 cells are deficient in N-sulfation of heparan sulfate chains and also show a lower general degree of heparan sulfate sulfation. pgsF-17 cells are deficient in 2-O-sulfation of heparan sulfate chains, but show relatively unchanged overall sulfation of heparan sulfate. (**E**) Internalization of pHrodo-α-synuclein fibrils in CHO K1 cells stably transduced with *HS3ST1-4*.

To investigate the specificity of the interaction, we took advantage of available mutant Chinese hamster ovary (CHO) cell lines altered in GAG biosynthesis. Wild-type CHO cells internalized pHrodo-α-synuclein amyloid fibrils readily (Fig. 6A), and treatment with heparin lyases and chondroitinase ABC showed that uptake depended strongly on heparan sulfate (Fig. 6B). Analysis of CHO pgsA-745, which lacks xylosyltransferase activity and therefore fails to make both heparan sulfate and chondroitin/dermatan sulfate, did not internalize α-synuclein fibrils (Fig. 6B). Transfection of pgsA-745 cells with *XylT1* restored uptake, which remained sensitive to heparin lyase digestion. Additionally, CRISPR/Cas9 was used to create two additional mutants defective in *XylT2*, the endogenous xylosyltransferase in CHO cells. These cells showed a similar reduction in uptake of α-synuclein fibrils compared to clonal wildtype lines (Fig. 6C). pgsD-677 cells showed greatly reduced uptake of α-synuclein fibrils. This mutant does not make heparan sulfate due to a deficiency in *Ext1*, a subunit of the copolymerase complex, but makes more chondroitin/dermatan sulfate proteoglycans (Fig. 6D)^37^. Thus, these mutants confirm the dependence of uptake of α-synuclein fibrils on heparan sulfate.

The CHO pgsE-606 line is deficient in glucosamine *N*-sulfation, which also lowers the overall degree of O-sulfation in heparan sulfate due to coupling of downstream O-sulfation reactions to N-sulfation of glucosamine units. Uptake of α-synuclein fibrils in pgsE-606 cells was reduced to similar levels as in pgsD-677 cells. In contrast, inactivation of 2-O-sulfation of uronic acids in pgsF-17 cells had only a small effect on uptake (Fig. 6D). Interestingly, this line produces heparan sulfate with elevated levels of N-sulfation and 6-O-sulfation, yielding chains with comparable overall charge to heparan sulfate made in wildtype cells. Thus, binding and uptake appears to be sensitive to overall charge of the chain, but independent of 2-O-sulfation per se. Introduction of 3-O-sulfate groups into heparan sulfate by transduction of wildtype CHO cells with four different isoforms of HS3ST enzymes also had little effect on α-synuclein fibril uptake (Fig. 6E).

## Discussion

It is becoming clear that the relationship between GAGs and amyloid-forming proteins is very intricate and complex. Interactions between these two classes of molecules have been described to have consequences for multiple processes involved in amyloid-related pathology^38^. This diverse role of GAGs in protein aggregate diseases makes it an attractive target for therapy. However, both detrimental and beneficial effects by GAGs on the disease development have been reported^22, 25, 39–43^. Although heparan sulfate mimetics have shown promising results as therapeutics in animal models^44–46^, clinical trials have been less successful. Thus, it is of importance to understand the molecular determinants that underlie GAG-amyloid interactions.

In this report, we have shown that neuronal binding and uptake of α-synuclein amyloid fibrils depends on cell surface heparan sulfate, presumably by binding to plasma membrane proteoglycans bearing heparan sulfate chains. Heparan sulfate proteoglycans also seem to facilitate neuronal internalization of other amyloid forming proteins, for example Aβ and tau (in Alzheimer’s disease) and prion protein (in prion diseases, for example Creutzfeldt-Jacob disease)^27–29^. In a recent study, a role for heparan sulfate in uptake of α-synuclein fibrils in neural precursor cells has also been suggested^30^. The general participation of heparan sulfate in several protein aggregation diseases reflects the propensity of various amyloid proteins to interact with polyanionic compounds, and the well-known capacity of cell-surface heparan sulfate proteoglycans to act as endocytic receptors^47, 48^.

In order to determine if the aggregate conformation, especially the amyloid fold, was of importance in binding and uptake, we studied two different aggregate conformations of α-synuclein - Thioflavin T-negative soluble oligomers and insoluble fibrillar aggregates with typical amyloid properties. In neuroblastoma cells, amyloid fibrils were taken up much more readily than the non-amyloid oligomers, indicating that the cells have a specific mechanism to interact with amyloid fibrils. Fibril formation might expose positively charged domains that can interact with heparan sulfate or create enhanced valency. Today, much focus is being put on oligomeric species, as they are believed to be more toxic than the fibrils^41^. However, it is possible that propagation and toxicity are caused by different conformational species, as has been proposed for prion disease^49^. Sonicated amyloid fibrils have repeatedly been shown to be able to seed aggregation of α-synuclein in cell culture as well as animal models^11, 14, 17, 33, 34^, and may therefore be responsible for propagation of pathology. Our findings suggest that such species would transfer between neuronal cells more effectively than smaller oligomers, as they seem to internalize more readily, although other oligomeric conformations than those used in this study need to be investigated as well. It should be noted that the relative affinity for fibrils compared to oligomers of the ELISA used in this study, has not been assessed. The signal acquired from fibrillar α-synuclein may therefore not be proportional to the signal acquired from oligomeric α-synuclein.

The oligomeric species of α-synuclein used in our study did not bind to cells through heparan sulfate. This finding is in contrast with Aβ, where amyloid fibrils, smaller oligomers, and even monomers have been reported to be dependent on heparan sulfate for internalization^28, 29^. Future studies should aim at determining if this difference is specific for the oligomeric preparation used in the present study, or if there are variations between different amyloid-associated proteins.

Discriminating between material that has been internalized and material that is only bound to the cell surface can be problematic, and in some previous studies on cellular uptake of protein aggregates this has been overlooked. Methods such as western blot on cell lysates or fluorophore-labeling of the aggregates with subsequent microscopy analyses have been employed, which each poses difficulties in determining what is truly internalized as opposed to attached to the outside of the cell. This prompted us to develop an assay to assess internalization using pHrodo-labeled α-synuclein. Consistent with previous findings suggesting that α-synuclein is shuttled through the endo-lysosomal pathway after internalization^50^, a time-dependent increase in the pH-induced fluorescence of pHrodo-tagged α-synuclein was observed. Uptake was sensitive to heparin lyases and heparin, but it is possible that the extent of internalization is underestimated with this method, due to turnover, entry by some other mechanism or if aggregates escape the endo-lysosomal pathway before sufficient acidification of the lumen occurs. However, comparable results were obtained by ELISA, which measures all α-synuclein regardless of its location within the cell or on the cell surface.

Although most reports on GAGs in amyloid deposits focus on heparan sulfate, chondroitin sulfate has also been described to be present in Lewy bodies as well as the Alzheimer’s disease related amyloid deposits of Aβ and tau^51, 52^. The findings reported here shows that heparan sulfate is the main GAG involved in cellular uptake of α-synuclein amyloid fibrils. Removal of chondroitin/dermatan sulfate had a mild effect on uptake in CHO cells, but no effect in the neuroblastoma or glial cell lines investigated in this study. Further studies of chondroitin sulfate are warranted, as different isomers of chondroitin sulfate occur in the brain, including species containing disulfated disaccharides^53^.

Studies have shown that α-synuclein aggregates can activate astrocytes and microglia, and that this appears to be dependent on aggregate internalization^54, 55^. Our study suggests that HS-dependent uptake of α-synuclein fibrils is used by non-immune brain cells like neurons and oligodendrocytes, while this pathway may be of less importance for astrocytes and microglial cells. Additional studies of primary cells and other cell lines are needed to confirm these differences. If correct, then therapeutic inhibition of heparan sulfate-dependent internalization would mostly affect transfer of amyloid aggregates between neurons (in Parkinson’s disease and Dementia with Lewy Bodies)) and oligodendrocytes (in Multiple Systemic Atrophy), while clearance of aggregates by microglial would largely be unaffected. As heparin is known to facilitate fibrillization of α-synuclein^25^ and other amyloidogenic proteins^22–24, 26^, it can be speculated that internalizing fibrils through cell surface HS, may pose a risk for continued aggregation in the recipient cell. In light of this, it can also be speculated that neuronal subtypes that express high levels of HS, or highly sulfated variants of HS, may be particularly affected in Lewy body disorders, due to both a high level of uptake and an increased risk for continued intracellular aggregation.

As heparan sulfate proteoglycans are ubiquitous and have a plethora of functions, a non-specific downregulation of these molecules to hinder cell-to-cell transfer of aggregates would likely be problematic. Therefore, the particular elements required for the interaction between heparan sulfate and the amyloid fibrils need to be elucidated. We used CHO mutants that are deficient in different aspects of heparan sulfate synthesis to study the interaction. The results suggest that the general degree of sulfation is important for the uptake, rather than a specific disaccharide sequence with certain modifications at particular locations.

In conclusion, we have shown that α-synuclein aggregates with an amyloid fibrillar fold seem to be highly dependent on cell surface heparan sulfate for internalization into non-immune cells of the brain, while additional mechanisms for internalization seem to be employed by astrocytes and microglia. We have also shown that a typical amyloid fibril structure seems to be important for the interaction between cell surface heparan sulfate and α-synuclein aggregates. These results warrant further studies to examine the involvement of heparan sulfate in disease propagation in Lewy body diseases *in vivo*.

## Materials and Methods

### Aggregation and labeling of α-synuclein

Human recombinant α-synuclein (rPeptide, Bogart, GA, USA) was used to produce non-amyloid oligomers and amyloid fibrils. To prepare the oligomers, α-synuclein was dissolved in 7.5 mM Tris, 100 mM NaCl, pH 7.4 at a concentration of 70 µM (~1 mg/ml) and incubated without agitation at 37 °C for 16 hr followed by 6 hr at 56 °C. To prepare fibrils, α-synuclein was dissolved in PBS to a concentration of 140 µM (~2 mg/ml), and incubated at 37 °C with rotary agitation (400 rpm). The formation of amyloid fibrils was monitored by the amyloid binding compound Thioflavin T^ 56^. Samples were taken from the α-synuclein solution at different time points and diluted to 4.5 µM, Thioflavin T was added to a concentration of 20 µM and the resulting fluorescence was measured at excitation max of 430 nm and emission max at 485 nm using a microplate reader (Spectramax M3, Molecular Devices, Sunnyvale, CA, USA). A plateau in the Thioflavin T curve was reached after 10 days and the fibrillization reaction was then stopped. The fibril solution was centrifuged at 20,000 × g for 30 min to separate the insoluble fibrils from smaller soluble aggregates and/or any residual monomers. The pellet was re-dissolved in PBS to a concentration of 70 µM. The amount of α-synuclein in the pellet was estimated by subtracting the concentration of protein in the supernatant from the concentration of protein in the solution before fibril formation was started. Concentrations were determined by measuring absorbance at 280 nm using NanoDrop. The re-dissolved pellet was then either sonicated or labeled with fluorescent tags.

Labeling of α-synuclein with pHrodo or AlexaFluor594 was performed with microscale labeling kits according to the manufacturer’s instructions (Thermo Fisher Scientific, Waltham, MA, USA). Purifying the fibrils from unreacted dye was achieved by centrifuging the α-synuclein/dye solution at 20,000 × g for 30 min, reconstitution in PBS, followed by 3 more cycles of centrifugation and reconstitution in PBS. The fibrils, labeled or unlabeled, were sonicated before adding to cells, using a probe sonicator (550 Sonic Dismembrator, Fisher Scientific) at power 2.5 for 20 × 5 sec.

### Electron microscopy

Samples were adhered to 100 mesh Formvar and carbon coated grids for 10 min at room temperature. Grids were washed 3 × 1 min with deionized water, stained with 2% uranyl acetate (Ladd Research Industries, Williston VT) in water for 1 minute, dried and viewed using a Tecnai G2 Spirit BioTWIN transmission electron microscope equipped with an Eagle 4k digital camera (FEI, Hilsboro, OR, USA).

### Gel electrophoresis and western blotting

Samples were diluted in NuPAGE lithium dodecyl sulfate (LDS) sample buffer and NuPAGE sample reducing agent (containing dithiothreitol (DTT)) (Thermo Fisher Scientific) according to the manufacturer’s instructions and heated at 95 °C for 10 min. Samples were separated on a 4–12% Bis-Tris gel (Thermo Fisher Scientific) using a 2-(*N*-morpholino) ethane-sulfonic acid (MES) sodium dodecyl sulfate (SDS) running buffer with NuPAGE antioxidant reagent (containing N,N-dimethylformamide and sodium bisulfate) (Thermo Fisher Scientific) added to the cathode solution. Samples were transferred to a PVDF membrane (EMD Millipore, Billerica, MA, USA) followed by blocking of the membrane in bovine serum albumin and incubation with an anti-synuclein antibody FL-140 (Santa Cruz Biotechnology, Dallas, TX, USA), and thereafter incubation with an anti-rabbit antibody (donkey-anti-rabbit IRDye680LT, Li-Cor, Lincoln, NE, USA) and analyzed with the Odyssey CLX system (Li-Cor).

### Cell culture and treatment

Cells were cultured at 37 °C and 5% CO_2._ Rat neuroblastoma B103 cells, human hybrid oligodendrocytic MO3.13 cells and murine microglial BV-2 cells were grown in DMEM medium (Gibco, Thermo Fischer Scientific), whereas Chinese Hamster Ovarian (CHO) cells and astrocytic-like rat glioma C6 cells were grown in F12 medium (Gibco, Thermo Fischer Scientific). Each medium was supplemented with 10% FBS (Gemini Bio, West Sacramento, CA, USA), 100 U/mL of penicillin and 100 μg/mL of streptomycin sulfate (Gibco, Thermo Fisher Scientific). All experiments shorter than 24 h were carried out in medium without FBS or antibiotics, whereas the concentration of FBS was reduced to 5% for longer experiments.

The production and characterization of CHO mutant pgsA-745 was described in ref. 57, pgsD-677 in ref. 37, pgsE-606 in ref. 58 and pgsF-17 in ref. 59. pgsA-745 cells transfected with xylosyltransferase 1 (pgsA-745-XT1) were described in ref. 60. New CHO cells bearing defects in xylosyltransferase 2 were created using CRISPR/Cas9 as described in ref. 61. CHO cells stably transduced with *HS3ST1-4* were created as described in ref. 62.

Alpha-synuclein was added to the culture medium at a concentration corresponding to 0.5 µM for monomeric α-synuclein. GAGs (heparin or chondroitin sulfate) were added to the cell media 5 min prior to the addition of α-synuclein, while GAG degrading enzymes (heparin lyases or chondroitinase ABC) were added 30 min prior to the addition of α-synuclein and re-added after 3 hr. Heparin (Scientific Protein Laboratories (SPL), Waunakee, WI, USA), chondroitin sulfate (shark cartilage chondroitin sulfate sodium salt, Sigma-Aldrich, Saint Louis, MO, USA) and chondroitinase ABC (AMSBIO, Cambridge, MA, USA) was obtained commercially, while recombinant heparin lyases were produced in *E*. *coli*.

### Alpha-synuclein sandwich ELISA

Cells were treated with α-synuclein for 4 hr and harvested with trypsin (0.25%), centrifuged and solubilized in PBS containing 1% Triton-X100 and a protease inhibitor cocktail (Complete, EDTA-free, Roche, Indianapolis, IN, USA). After centrifugation at 20,000 × g for 30 min, the resulting supernatant and pellet were separated and the pellet re-dissolved in PBS containing 1% Triton-X100 and 1% SDS using a probe sonicator (550 Sonic Dismembrator, Fisher Scientific) at power 2.5 for 15 seconds followed by heating at 75 °C for 10 min. The samples were then analyzed for α-synuclein levels using a sandwich ELISA with minor changes from the procedure described in ref. 63. In short, high-binding 96-well EIA/RIA plates (Corning Inc., Corning, NY, USA) were coated with anti-Syn-1 antibody at 0.3 µg/ml (BD Biosciences, San Diego, CA, USA) at 4 °C overnight. Plates were blocked with 1% bovine serum albumin in PBS for 3 hr in room temperature before samples were applied for 1 hr at room temperature. For detection, an anti-synuclein antibody (FL-140, Santa Cruz Biotechnology) was used at a concentration of 0.3 µg/ml, followed by incubation with an HRP-conjugated goat anti-rabbit antibody diluted 1:5000 (Vector Laboratories). Enhanced K-Blue TMB substrate (Neogen, Lansing, MI, USA) was added and the reaction was stopped with 1 M HCl and the absorbance at 450 nm was measured using a microplate reader (Spectramax M3, Molecular Devices). All experiments were repeated at least three times.

### Flow cytometry

Cells were treated with pHrodo-labeled α-synuclein fibrils for 8 hr and harvested with trypsin, centrifuged, resuspended in a solution of 1 mM EDTA in PBS and immediately analyzed by flow cytometry (BD FACSCalibur). CellQuest software was used to analyze the acquired data. Each condition was performed in triplicate, and 10000 cells from each well were analyzed.

### Confocal microscopy

Cells were treated with AlexaFluor594-labelled α-synuclein fibrils before being washed 3 times in PBS and fixed with 4% paraformaldehyde at different time-points. Cells were made permeable with 0.25% Triton-X100, blocked with 10% horse serum (Vector Labs, Burlingame, CA, USA), stained with anti-heparan sulfate antibody 10E4 (AMSBIO) at a dilution of 1:100, followed by staining with fluorescein-conjugated secondary antibody (1:100, Vector Labs). Slides were mounted with ProLong® Gold Antifade Mountant (Thermo Fisher Scientific) and studied with a Axiovert 35 microscope (Zeiss, Germany) with an attached MRC1024 laser scanning confocal microscope system (BioRad, Hercules, CA, USA) and analyzed with Image J v1.43 software (NIH, Bethesda, MD, USA).

### Statistical analyses

All values are shown as mean values ± standard deviation. Tests for significance were performed with one-way ANOVA with Sidak’s multiple comparisons test or Student’s t-test. *P* ≤ 0.05 was considered significant. *P ≤ 0.05, **P ≤ 0.01, ***P ≤ 0.001.
